# Protein–protein interaction prediction methods: from docking-based to AI-based approaches

**DOI:** 10.1007/s12551-022-01032-7

**Published:** 2022-12-17

**Authors:** Yuko Tsuchiya, Yu Yamamori, Kentaro Tomii

**Affiliations:** Artificial Intelligence Research Center (AIRC), National Institute of Advanced Industrial Science and Technology (AIST), 2-4-7 Aomi, Koto-Ku, Tokyo, 135-0064 Japan

**Keywords:** Protein–protein interaction, Antibody–antigen interaction, Epitope prediction, Docking simulation, AlphaFold, Profile–profile comparison

## Abstract

Protein–protein interactions (PPIs), such as protein–protein inhibitor, antibody–antigen complex, and supercomplexes play diverse and important roles in cells. Recent advances in structural analysis methods, including cryo-EM, for the determination of protein complex structures are remarkable. Nevertheless, much room remains for improvement and utilization of computational methods to predict PPIs because of the large number and great diversity of unresolved complex structures. This review introduces a wide array of computational methods, including our own, for estimating PPIs including antibody–antigen interactions, offering both historical and forward-looking perspectives.

## Introduction

Protein–protein interactions (PPIs) play fundamentally important roles in cellular functions and biological processes, and structural understanding of the PPIs is important for the elucidation of those functions (Jones and Thornton 1995, 1996). In 2001, the Critical Assessment of PRediction of Interactions (CAPRI, 2022) began as a community-wide experiment designed to assess methods for predicting PPIs based on the estimation of PPIs for previously solved structures of protein complexes. The latest experiment (Round 54) was conducted during May–August in 2022 (CAPRI Round 54, 2022). A recent report of a CAPRI experiment (Lensink et al. 2020) indicated that the increase of structures of protein complexes deposited in the Protein Data Bank (PDB) enables their use as structural complex templates for predicting the structures of other protein complexes, particularly in homo protein–protein docking. That finding implies that classical docking may no longer be strictly necessary for the prediction of homo PPIs. Nevertheless, these methods have not yet been successfully applied to the prediction of hetero PPIs, including antibody–antigen interactions, and this area of research has room for improvement. In this review, we place our focus on computational docking-based approaches and AI-based approaches, introducing a wide array of methods, including our own, for PPI prediction.

## Protein–protein docking

Traditional protein–protein docking methods have been of central importance for sampling the conformational space of protein complexes (Smith and Sternberg 2002). In the last 10 years, sophisticated high-precision docking methods such as HADDOCK (van Zundert et al. 2015), ClusPro (Desta et al. 2020), ZDOCK (Pierce et al. 2014), and LightDock (Jiménez-García et al. 2018) have been developed and continually improved. To sample conformations more efficiently, they have used not only three-dimensional structural information in both modeling and scoring steps, but also evolutionary information and information obtained from experiments of several types (van Noort et al. 2021). Evolutionary information has been used for the prediction of PPIs based on the idea that mutations of the residues involved in the interaction on a protein engender mutations of the interface residues on the partner protein (Jothi et al. 2006). The InterEvDock docking pipeline integrates a coarse-grained potential accounting for interface coevolution based on multiple sequence alignments (MSAs) of paired proteins (Yu et al. 2016). For many targets in recent CAPRI experiments, this method predicted several high-quality models (Lensink et al. 2020).

Professor Nakamura and his colleagues, including one of the authors of the present review (Y.T.), participated in CAPRI about 10 years ago (Fleishman et al. 2011; Moretti et al. 2013; Lensink et al. 2014). For each CAPRI experiment target, the docking of two component (mainly unbound) protein structures was performed to generate many complex models, and the position and relative geometry of the predicted interfaces were evaluated based on the scoring function. We constructed docking methods with and without evolutional tracing using conservation information, where the conservation information contributed to model selections particularly for the targets of enzyme and signal transduction proteins (Kanamori et al. 2007).

Recently, data of low-resolution shapes obtained by small-angle scattering methods such as SAXS, were also used for the filtering of docked models in several docking methods (van Noort et al. 2021), e.g., pyDockSAXS (Jiménez-García et al. 2015) and HADDOCK (van Zundert et al. 2015).

## Scoring models

After sampling complex models, the most “appropriate” model(s) must usually be selected using some method such as scoring function, clustering, or consensus (Lensink and Wodak 2010). In CAPRI, the “scorer” performance is measured and reported as part of the competition (Lensink and Wodak 2010). Predicted models for each target were pooled and provided for the scoring experiment. Based on the idea that the accumulation of complementary interactions on local (residue or atomic level) surface regions forms the PPI between two proteins and strengthens their interaction, we specifically examined the complementarities of physicochemical properties such as electrostatic potential and hydrophobicity, and shapes of the surfaces to assess PPIs on the molecular surfaces of proteins (Tsuchiya et al. 2006a, b).

Machine-learning (ML) and AI-based scoring methods have been developed in the past few years (Lensink et al. 2020). The ML-based iScore is a linearly combined score of GraphRank with HADDOCK energetic terms (Geng et al. 2020). The GraphRank score is based on a support vector machine (SVM) classification between the interface graphs of native and non-native protein–protein interfaces, where the graph consists of nodes (interface residues with evolutionary conservation scores) and edges (their contacts) (Geng et al. 2020). The iScore separated native from non-native interfaces much better than the case of individual usage of the GraphRank or HADDOCK score, when the GraphRank contribution is higher than the energetic terms. Das et al. also developed an SVM classification-based scoring scheme by particularly addressing differences between native and non-native protein–protein interface features such as binding energy (ΔG), frequencies of hydrogen bonds and salt-bridges, and accessible and buried surface areas (Das and Chakrabarti 2021). Their findings demonstrated the accessible surface area as the most distinguishable feature. In native interfaces, the buried surface areas of Phe, Tyr, and Ile are markedly high whereas that of Lys is low, and hydrogen bonds in Arg with Asp or Glu are more important (Das and Chakrabarti 2021). Scoring based on these features led to higher accuracy than that provided by PatchDock scores (Schneidman-Duhovny et al. 2005). Additionally, we have developed the Protein Interface Analysis using COvarying signals (PIACO 2019), a method for identifying biological interfaces in protein crystal structures. PIACO is a statistical classifier that uses covariation calculated from MSAs as input (Fukasawa and Tomii 2019).

Another recently developed AI-based scoring method, known as DOVE, is based on the use of three-dimensional (3D) convolutional neural networks (Wang et al. 2020). It first maps the inputted decoy structures into a 3D grid, then scans and examines the protein–protein interfaces in terms of inter-atom interaction patterns and their energetic contributions. Finally, DOVE judges whether the decoy structure is close to the native structure, or not.

## Hot spots

In PPI prediction, a hot spot is a prominent feature that allows one to distinguish between native and non-native interface models (DeLano 2002). Typically, a hot spot is a small set of residues that contribute greatly to PPI formation (Thorn and Bogan 2001). From the converse perspective, blocking hot spot residues might engender the disruption of the PPIs (Lu et al. 2020). Hot spots are fundamentally identified based on changes in binding free energy (ΔΔG) obtained by alanine scanning mutagenesis, whereas computational methods of the accurate predictions of hot spots have also been developed (Ovek et al. 2022). Ovek et al. introduce two interesting examples in their recent review (Ovek et al. 2022): disease-causing single mutations are likely to occur on hot spots (Ozdemir et al. 2018) and disease-causing PPI can be disrupted by a small molecule that binds to the target hot spots on the PPI (Lim et al. 2019). These findings suggest that hot spot information might contribute to the discovery of drugs that disrupt the disease-causing PPI (Ovek et al. 2022).

When we participated in the CAPRI experiment, several groups tried to predict hot spots to select near native models. Of those, Fernandez-Recio’s group predicted hot spots using the normalized interface propensity values derived from rigid-body docking, with electrostatics and desolvation scoring, which required no structural information of protein–protein complexes (Grosdidier and Fernández-Recio 2008). The same group recently constructed a method to detect protein–protein inhibitor (small molecule) binding sites by integrating molecular dynamics simulations for the generation of transient cavities on the interface with hot-spot predictions (Rosell and Fernández-Recio 2020). It generated and selected the transient cavities that were similar to known inhibitor binding sites (Rosell and Fernández-Recio 2020).

## Template-based method

As we mentioned, protein–protein docking has been centrally important to PPI prediction. However, along with the increase of solved complex structures, it has been found to be more helpful to use them as templates for PPI prediction. As mentioned earlier (Lensink et al. 2020), found that the use of solved complex structures as templates is particularly appropriate for homo PPI prediction because of the abundance of such homo PPI structures within the PDB.

As an example, we introduce work from our own group in which we have developed a profile–profile comparison method: Fold Recognition TEchnique (FORTE) (Tomii and Akiyama 2004). The method was developed originally for predicting protein structures. We recognized that detecting and using complex templates is effective for PPI prediction (even for hetero PPI in some cases) through participation in past CAPRI-CASP assembly prediction experiments (Lensink et al. 2016, 2018; Nakamura et al. 2018). We also found that FORTE is helpful for selecting an appropriate form of the protein complex, even for cases with multiple forms of complexes observed among homologous proteins of a target (Nakamura et al. 2018). Indeed, as we mentioned below, most of AI-based approaches which utilize information of templates are powerful for PPI, except in the case of antibody–antigen interaction prediction (Ambrosetti et al. 2020).

As a general rule, the prediction capability for generating accurate target structures depends on the existence of available templates in the PDB (Lafita et al. 2018). In CAPRI-CASP12, according to the assessors’ definition, there were three levels of difficulty for estimating target complexes—EASY, MEDIUM, and HARD. We showed that template-based models based on profile–profile comparison methods are useful for predicting protein complexes, even for MEDIUM/HARD targets (Nakamura et al. 2018; Lafita et al. 2018; Lensink et al. 2018). This finding implies that PPIs also tend to be conserved and/or limited for many cases, although it is considered that complex structures are often not conserved during evolution (Poupon and Janin 2010).

## Earlier attempts of application of AlphaFold2 to PPI prediction

When the great achievement of AlphaFold2 (AF2) for the modeling of monomeric structures of protein in CASP14 was announced, it was assumed that AF2 would be useful for the modeling of protein–protein complexes, even though it was originally trained on individual protein chains (Jumper et al. 2021). RoseTTaFold, the first attempt to replicate AF2 before the release of the AF2 codes, confirmed that deep neural network model trained for monomer structure prediction can predict protein–protein complex models with some degree of accuracy (Baek et al. 2021). They used the pseudo-multimer sequence, two or more sequences with a gap separating them, (and templates) as input instead of a monomer sequence. AF2 and RoseTTaFold have since been used for predicting PPIs in proteomes of human (Burke et al. 2022) and yeast (Humphreys et al. 2021).

Similar tricks to that developed in RoseTTaFold were also applied for AF2, such as AF2-Gap (in ColabFold (Mirdita et al. 2022)) and AF2-Linker (Moriwaki 2021; Evans et al. 2021). Other groups applied similar protocols for protein–peptide docking (Ko and Lee 2021; Lei et al. 2021; Tsaban et al. 2022). All of these used the pseudo-multimer sequence linking the peptide to the protein via poly-glycine as input. The protocol that was tested for the benchmark set shows higher performance than the previous state-of-the-art method (Tsaban et al. 2022). FoldDock, another sophisticated AF2-based multimer prediction method using a paired MSA, was developed and tested for a large benchmark set of heterodimers (Bryant et al. 2022a). These results underscore the superiority of AF2-based methods to other docking methods. Although the method described above is powerful for dimers (or trimer, perhaps), they might have limitations because of the difficulty in preparing good MSAs of larger complexes as their input. In contrast, MolPC is an ambitious attempt for using AF2 to build a larger complex (more than 10 chains) combining the predicted subcomponents using Monte Carlo tree search (Bryant et al. 2022b). The AF2Complex uses the MSAs of each chain by padding the gaps and templates as inputs and by generating complex models by the AF2 deep learning model after multiple recycling steps (Gao et al. 2022).

A combination of AF2 and an existing docking method was also tested (Ghani et al. 2021). The AF2-ClusPro method was adopted for the protein–protein docking benchmark 5.0 (BM5) (Vreven et al. 2015). Then we selected 17 heteromers which appeared in the PDB after May 2020 (the last date of structures utilized in the AF2 training set). The systematic benchmark of 152 diverse heterodimers from BM5.5 data set (Vreven et al. 2015; Guest et al. 2021) was expanded by adding more antibody–antigen cases to BM5, with results revealing a significant ability of AF2-based methods for all categories of modeling of complexes (except for modeling of antibody–antigen complexes) (Yin et al. 2022), approaches represented early attempts for AF2-based multimer prediction done mainly during the period between the release of the original AF2 (ver. 2.0.0) and that of the specifically trained AF2 for multimer prediction (ver. 2.1.0).

## Multimetric version of AF2 and recent attempts to go beyond AF2

The multimeric version of AF2, AF2-Multimer, appeared recently (Evans et al. 2021). Benchmarking results obtained for the selected 17 heteromer and a large data set composed of recently released 4433 complexes was compared with AF2-Gap, AF2-linker and AF2-ClusPro, and it showed improvement in multimer prediction accuracy compared with those input-adjustment AF2-based multimer predictions. It was also demonstrated that the performance is generally higher for homomeric prediction than for heteromeric prediction, and that it is poor for prediction of the antibody–antigen complex. The latter result was also clarified by the benchmark of another group (Yin et al. 2022).

More recently, two avenues of approaches were used to go beyond the AF2 and AF2-Multimer which involved the development of replication of AF2 with a larger training dataset and development of the predictor using the protein language model (pLM) instead of MSA. Among the follow-up replicative studies of AF2, such as OpenFold (Ahdritz et al. 2022), Uni-Fold (Li et al. 2022a, b), MegaFold (Liu et al. 2022), and HelixFold (Wang et al. 2022), it was only Uni-Fold and Uni-Fold-symmetry (Li et al. 2022b) that could succeed not only in monomer prediction but also in multimer prediction upon using the original trained parameters or protocols. No third-party group has yet published a large benchmark result. Actually, pLM-based predictors such as OmegaFold (Wu et al. 2022), ESMFold (Lin et al. 2022), and IgFold (Ruffolo et al. 2022) are anticipated as the next breakthroughs in structure prediction because they omit the construction of MSA, which is crucially important for performance, and which is the most time-consuming part of AF2. In spite of these expectations, no MSA-free method has achieved performance equal to that of AF2, with no explicit implementation of multimer modeling with the exception that IgFold was built specifically for antibody modeling.

**Fig. 1:**
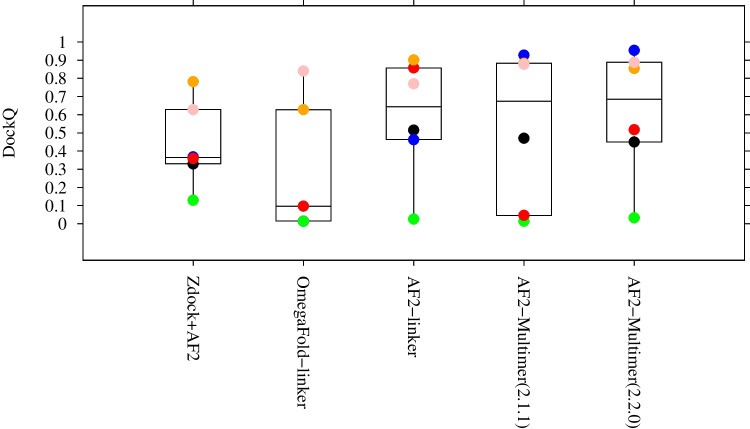
Distributions of DockQ scores as boxplots for different modeling methods: ZDOCK with AF2 models, OmegaFold-linker, AF2-linker, AF2-Multimer (v.2.1.1), and AF2-Multimer (v2.2.0). Six targets (T1032 (shown in black dot), T1038 (blue), T1054 (green), T1078 (red), H1045 (orange), and H1065 (pink)) are taken from CASP14 (see text). For the case of OmegaFold-linker, T1032 was omitted because its amino acid length is beyond the limitation of OmegaFold

Although no explicit implementation of multimer treatment in OmegaFold has been described, it is relatively straightforward to use pseudo-multiple sequence inputs as an AF2-linker which we designate as OmegaFold-linker. Figure 1 presents multimer modeling results in terms of the DockQ score (Basu and Wallner 2016) using methods developed post AF2 such as OmegaFold-linker, AF2-Multimer (v2.1.1) with parameters released on Nov. 2021, and AF2-Multimer (v.2.2.0) with parameters released on Mar. 2022. In the construction of Fig. 1, AF2 was run with “max_template_date = 2020–10–01”. The targets are taken from those of CASP14 (not included in the training set of AF2 and AF2-multimer, but two of these structures (PDB IDs: 6N64 and 6YA2) of them are released before 2020–10–01). They are all dimers, and the target IDs are T1032 (PDB ID: 6N64), T1038 (PDB ID: 6YA2), T1054 (PDB ID: 6V4V), T1078 (PDB ID: 7CWP), H1045 (PDB ID: 6XOD), and H1065 (PDB ID: 7M5F). For OmegaFold-linker, due to the memory-limitation, T1032 with more than 500 residues was omitted. For Omega-linker and AF2-linker, the input sequences were connected via 21 length poly-GLY-GLY-SER linker according to the previous research (Evans et al. 2021). For comparison, DockQ scores of docking structures calculated using ZDOCK (Pierce et al. 2014) are also shown. As the initial monomer structures of ZDOCK, the same models predicted by AF2-Multimer (v.2.2.0) were used. Basically, the prediction difficulty depends on targets. For instance, all methods except ZDOCK show 0.77 or more for H1065, while all methods show DockQ < 0.04 for T1054. These trends, except for T1038 (6YA2) whose structure was released before 2020–10–01, are generally similar to those observed in CASP14. Although, this may imply that the prediction difficulty of PPI depends on the availability of “good” templates, the results accord with those reported from benchmark research (Evans et al. 2021; Bryant et al. 2022a): AF2-Multimer shows the highest performance, and AF2-based methods using parameters trained for monomers are still comparable in some cases.

## Antibody–antigen interaction

The prediction of PPI between antibody and antigen proteins is not an easy task because of the flexibility of the antibody’s hypervariable loops, particularly the complementary determining loop 3 in the heavy chain (CDR-H3 loop). Four high-precision docking software suites ClusPro (Brenke et al. 2012), LightDock (Jiménez-García et al. 2018), ZDOCK (Pierce et al. 2014), and HADDOCK (van Zundert et al. 2015) were used to examine which structural information contributes to the accuracy of the model building of an antibody–antigen complex, such as the structural information of CDR loops, paratope (antigen-binding residues on an antibody CDR), antigen surface, and epitope (antibody-binding residues on an antigen) (Ambrosetti et al. 2020). The findings showed for all docking methods that the overall performance decreased without epitope information. They were improved by consideration of the low-resolution epitope information. Accurate modeling of the structure of the long CDR-H3 loop remains challenging. However, the flexible refinement of HADDOCK led to the improvement of the prediction accuracy of CDR-H3 loop conformations if the epitope information was available, even though the resolution of the information is low (Ambrosetti et al. 2020).

The AI-based prediction of antibody–antigen interfaces has been developed as PECAN (Pittala and Bailey-Kellogg 2020). Here, antibody and antigen structures are presented as the respective graphs. They are input to the neural network that consists of graph convolution, attention, and fully connected layers. The network discriminates antibody and antigen residues between interface and non-interface residues. The predictive accuracies of PECAN achieved when using the datasets provided with epitope (Krawczyk et al. 2014), and paratope prediction methods (Daberdaku and Ferrari 2019) were found to have higher precision and recall rates than these providers’ methods. It is noteworthy that the providers’ methods also predict epitope and paratope regions with high accuracies. Epitope prediction, using the program EpiPred, predicts epitopes based on geometric fitting and knowledge-based asymmetric antibody–antigen scoring (Krawczyk et al. 2014). The paratope prediction method uses a SVM classifier to distinguish interface surface patches from non-interface ones based on 3D Zernike descriptors that represent global and local protein surface shapes and physicochemical properties on the surfaces (Daberdaku and Ferrari 2019). Again, note that even recent AI-based approaches, such as AF2-Multimer, suffer from an inability to predict accurate antibody-antigen complex structures (as mentioned above).

To obtain information about antibody-specific epitopes, some improvements in the accuracy of docking and affinity predictions must be achieved. For this purpose, a large and non-redundant benchmark set for antibody–antigen docking and affinity prediction was constructed. It includes camelid nanobodies, therapeutic monoclonal antibodies, and broadly neutralizing antibodies that target viral glycoproteins (Guest et al. 2021).

Another possible approach is the search of similar regions on proteins to known antibody-binding epitopes. This approach is based on the fact that some antibodies can cross-reactively recognize different antigen proteins having similar surface regions in the structures and properties (Vieths et al. 2006; Negi and Braun 2017). Information about cross-reactivity might provide information about the repurposing of antibody drugs. We are striving to develop a database of known and putative epitopes on proteins (PoSSuMAg 2022), which has a similar scheme to that of the PoSSuM database (PoSSuM 2021), which is helpful to detect putative pockets that are similar to known ligand-binding sites on protein structures (Tabei et al. 2010; Ito et al. 2012b, a, 2015). Our new database presents information about putative epitopes that are similar to known epitopes on the antigen proteins in complex with antibodies, including antibody drugs. Information about known and putative epitopes on SARS-CoV-2 proteins will also be available. Although the current version includes information of putative epitopes on non-redundant protein structures, we expect to increase the data through future studies.

## Concluding remarks

Here we have introduced and discussed a diverse range of computational methods, from docking-based to AI-based approaches, for PPI prediction. Along with the increase in the amount of information related to protein sequences and structures, AI-based approaches, especially those based on evolutionary information and templates, are expected to become more powerful and useful. Yet, prediction of hetero PPIs leaves room for improvement. Particularly, antibody–antigen interaction predictions remain very limited in terms of their accuracy, although many sophisticated prediction methods have been developed (Ambrosetti et al. 2020).

## Data Availability

All the data associated with Fig. 1 are available from our figshare repository (Yamamori et al. 2022).
